# Implementation of Universal Infant Free School Meals: a pilot study in NE England exploring the impact on Key Stage 1 pupil’s dietary intake

**DOI:** 10.1017/S1368980020004875

**Published:** 2020-12-02

**Authors:** Suzanne Spence, John NS Matthews, Lorraine McSweeney, Maisie K Rowland, Phoebe Orango, Ashley J Adamson

**Affiliations:** 1 Human Nutrition Research Centre, Population Health Sciences Institute, Faculty of Medical Sciences, Newcastle University, M1.151 William Leech Building, Framlington Place, Newcastle upon Tyne NE2 4HH, UK; 2 Fuse, The Centre for Translational Research in Public Health, Newcastle University, Newcastle upon Tyne, UK; 3 School of Mathematics, Statistics and Physics, Newcastle University, Newcastle upon Tyne, UK

**Keywords:** Pupils, School lunch, Universal Infant Free School Meals, Diet

## Abstract

**Objective:**

To consider the principal effect of an interaction between year (pre- and post-Universal Infant Free School Meals (UIFSM)) and school on pupil’s dietary intakes.

**Design:**

A repeated cross-sectional survey using dietary data from 2008 to 2009 (pre-) and 2017 to 2018 (post-UIFSM)

**Setting:**

Two primary schools, NE England.

**Participants:**

Pupils aged 4–7 years (2008–2009 *n* 121; 2017–2018 *n* 87).

**Results:**

At lunchtime, there was a statistically significant decrease in pupils non-milk extrinsic sugars intake (%E NMEs) pre- to post-UIFSM (mean change –4·6 %; 95 % CI –6·3, –2·9); this was reflected in total diet (–3·8 %; –5·2, –2·7 %). A year and school interaction was found for mean Ca intakes: post-UIFSM pupils in School 2 had a similar mean intake as pre; in School 1 intakes had increased (difference of difference: –120 mg; 95 % CI –179, –62); no reflection in total diet. Post-UIFSM mean portions of yogurt decreased in School 2 and remained similar in School 1 (–0·25; –0·46, –0·04); this was similar for ‘cake/pudding’ and fruit.

**Conclusions:**

Within the limitations, these findings highlight positives and limitations following UIFSM implementation and demonstrate the role of school-level food practices on pupil’s choices. To facilitate maximum potential of UIFSM, national levers, such as discussions on updating school food standards, including sugars, could consider removing the daily ‘pudding’ option and advocate ‘fruit only’ options 1 d/week, as some schools do currently. Small school-level changes could maximise positive health impacts by decreasing NMEs intake. A more robust evaluation is imperative to consider dietary impacts, equitability and wider effects on schools and families.

It is well established that school-age pupil’s diets require improvement^(1,2)^. There is also evidence that diets are affected by level of deprivation; individuals from more-deprived backgrounds have diets of a lower nutritional quality^(3–6)^. Many factors influence what school-age pupils eat and drink, including food preferences, parents, social activities and the environments they interact with, such as home, school and the wider out of home environment^(7,8)^. To improve what pupils eat and drink in schools, several legislative changes have been implemented to all state-funded primary schools in England in recent years. In September 2008, food- and nutrient-based standards were introduced in England^(9)^, these included specific requirements for what ‘types’ of foods could be served and frequency, along with minimum and maximum requirements for macro- and micro-nutrients over a 3-week menu cycle. Food-based standards included requirements for fruit and vegetables; starchy foods; foods high in fat, salt and sugar; meat, fish and eggs, and non-dairy sources (2017–2018 only). Nutrient-based standards required that an average school lunch provided specified amounts of energy, saturated fat, fat, sugars, protein, carbohydrate, Ca, Fe and a number of other micro-nutrients^(9)^. All state-funded primary schools in England were required to comply with this legislation; less focus was given to implementation of the policy in schools and monitoring compliance. Both schools included in this pilot study are maintained primary schools and therefore must comply with the food-based standards. In 2013, the School Food Plan was commissioned by the then government to review school food. As part of the School Food Plan, the 2008 food- and nutrient- based standards were changed to include food-based standards only and updated to include non-dairy items^(10)^. While all state-funded primary schools are required to comply with the food-based standards, there is potential for variation, for example, some school headteachers may choose to have a fruit only option 1 d a week compared with a daily cake/pudding option. A recommendation from the school food plan was to implement Universal Infant Free School Meals (UIFSM) to Key Stage 1 pupils (Reception to Year 2; aged 4–7 years) in England^(11)^. UIFSM were adopted from September 2014, in England^(12)^, with an allocated budget of £450 million.

Although prior research highlighted the potential positive impacts of food- and nutrient-based standards on pupils’ diets at lunchtime and total diet^(13,14)^, there has been no definitive quantitative evaluation of the implementation of UIFSM on pupil’s dietary intake since implementation in 2014. A small pilot study in Durham, a city in north east England, highlighted potential positive effects of implementing UIFSM on attainment and limited beneficial effects on dietary intake^(15)^. In 2015, Scotland moved from a targeted system of Free School Meals to all pupils in Primary 1 to 3 receiving Universal Free School Meals (UFSM)^(16)^. A process evaluation of UFSM implementation was undertaken with parents, schools and local authorities. Qualitative findings highlighted newly eligible families expressed positive views of a UFSM policy, for example, financial benefits and perceived nutritional impacts. Further, schools noted benefits and limitations, for example, increased food waste, staffing and dining capacity issues, and UFSM uptake was found to fluctuate between schools in Scotland^(16)^. Recently, Rabe *et al.* reported findings from their study exploring the effect of UIFSM on a number of outcomes, for example, take-up of school lunches, among newly eligible children and children that were previously in receipt of Free School Meals, and BMI^(17)^. Pre-UIFSM take-up among non-eligible children was just over 30 %, once UIFSM was implemented in 2014–2015 around 85 % of children were consuming them^(18)^. They found by the end of the academic year, on average, a child receiving a free school lunches is ‘1·2 percentage points more likely to be of healthy weight and 0·7 percentage points less likely to be obese’^(17)^. Rabe *et al*. propose UIFSM are potentially more successful than other school-based initiatives to reduce levels of obesity^(17)^. They perceive this effect is as a result of children not previously eligible for free meals taking them, suggesting that the diets of children from less deprived families can be improved by consuming school lunches^(18)^. This finding, that on average school lunches provide a more nutritionally balanced meal compared with home-packed lunches, is supported by prior research^(13,14,19)^. Although Holford *et al*. note that these findings are potentially due to dietary intake from school lunch^(18)^, the authors are not aware of any exploration of the impact of UIFSM on children’s actual dietary intakes. Within the limitations of a pilot study employing a natural experimental evaluation, we explore the impact pre- and post-UIFSM on pupil’s mean dietary intake of selected macro- and micro-nutrients, and key foods: fruit, vegetables, cakes, biscuits and sweet puddings. These key foods have been selected as on average children do not consume enough fruit and vegetables and consume too much sugar obtained from cakes, biscuits and sweet puddings. With UIFSM potentially providing a more nutritionally balanced meal, we would hypothesise average fruit and vegetables intakes increase and average intakes of cake, biscuits and sweet puddings decrease. We explored the effect of the UIFSM policy in two schools in NE England. In 2008–2009, School 1 had a higher take-up of school lunches, in comparison, School 2 had a mixed take-up of school and home-packed lunches, with substantially more pupils consuming a home-packed lunch. By 2017–2018, a large majority of pupils in both schools (School 1 and School 2) now consumed a school lunch due to the change in policy. Our key aim is to consider the principal effect of an interaction between year (pre- and post-UIFSM) and school (the ‘proxy’ measure to observe the effect of a change in lunch, i.e., school lunch or home-packed lunch) at lunchtime in two primary schools in NE England. Our secondary aim is to consider the effect on children’s total diet.

## Methods

### Study design, setting and participants

We employed a repeated cross-sectional survey over two academic years (2008–2009 pre-UIFSM) and (2017–2018 post-UIFSM). The survey data from 2008–2009 were collected as part of a study examining the implementation of the food- and nutrient-based standards on pupil’s diets in Reception, Year 1 and Year 2 pupils in NE England^(13)^. All surveys have employed the same methods, which are briefly detailed here.

As this was a pilot study, a convenience sample was used, two school Head Teachers from the same twelve schools who participated in the 2008–2009 research study were provided with the study information letter by email and asked if they would be willing to participate. This was followed up with a telephone conversation to clarify questions and arrange suitable dates for researchers to visit the school. A short talk was given in each school to the pupils in Reception to Year 2 to explain the study and show pupils the dietary data collection tool. Each pupil received a study information pack including a parental consent form requesting permission to participate. Consent forms were collected from the schools by the study researcher. The two schools were selected to include variation by level of deprivation, School 1 was located in a more deprived ward and School 2 in a less deprived ward. While this provides a measure of deprivation by school location, we used individual pupil-level demographics to consider the level of deprivation effect as explained in the statistical analysis section. In 2008–2009, School 1 had a higher take-up of school lunches, in comparison, School 2 had a mixed take-up of school and home-packed lunches, with substantially more pupils consuming a home-packed lunch. By 2017–2018, a large majority of pupils in both schools now consumed a school lunch due to the change in policy. As a token of appreciation, participating schools received a £1 voucher per pupil who participated.

### Data collection

The same dietary data collection method previously reported was used in the current study and is briefly described here^(13)^. A prospective 24 h food diary (FAST – the Food in Schools Assessment Tool) combining elements of a food diary and food frequency method was used to collect four consecutive days of pupils dietary data: Wednesday to Saturday. FAST has been previously validated against a 4-d weighed dietary intake of pupils aged 4–7 years to assess pupils food and drink consumption in the defined time periods, that is, 06.00–09.00^(20)^. This prior validation of FAST compared the 4 d weighed dietary intakes (WFI) collected in a crossover design from a subsample of participating children (*n* 70). Method comparison analysis included simple correlation, assessment of bias and limits of agreement. The findings for fruit, and fruit and vegetables have been previously reported as follows: for fruit, mean intakes of 2·1 and 2·0 portions/d (limits of agreement –0·6 to 0·5) and for fruit and vegetables combined 3·4 and 3·2 portions/d (limits of agreement –1·3 to 0·8 portions) were reported for WFI and FAST, respectively^(20)^.

Outcomes of key macro- and micro-nutrients were previously reported, for example, mean energy (kcals). Food and drink portions are age and sex specific and derived from the National Diet and Nutrition Surveys^(2)^. This dietary method for young children has been used in a number of studies exploring changes in young children’s diets^(13,21)^. School lunch information has been calculated using the same method as in 2008–2009^(13)^ and is derived from average portions based on the school recipe information obtained from the same Local Authority catering provider of each school.

Each parent received full written instructions on how to complete the food diary outside of school hours. During school hours, a team of researchers observed and recorded pupil’s dietary intake across the school day, this included breakfast clubs, break time, lunchtime and afterschool clubs. This enabled food and drink consumption to be analysed by different time periods. Dietary coding was based on McCance and Widdowson’s Integrated Composition of Food Dataset^(22)^. School recipes were obtained to code the nutrient composition of school foods.

Main outcome measures were change in mean daily intakes of: non-milk extrinsic sugars (NMEs) as percentage energy (%E), carbohydrates (%E), saturated fat (%E) and fat (%E); total energy (kJ), food weight (g), Ca (mg), Fe (mg) and vitamin C (mg) at both lunchtime and in total diet. Change in mean daily intakes of fruit, vegetables, biscuits, cakes and sweet puddings was also measured at lunchtime.

### Statistical analysis

The sample size of the study was limited by the fact this was a pilot study in two primary schools. Preliminary overall numbers are provided for the sample population including gender, level of deprivation (categorised by Index of Multiple Deprivation) and lunch type by school and year. Level of deprivation was estimated using the English Indices of Multiple Deprivation, 2015 and pupil-level postcodes^(23)^. This provides the Lower-layer Super Output Area that each postcode falls within, and the deprivation data for that Lower-layer Super Output Area enabling pupil-level data to be ranked by level of deprivation^(23)^. All pupil postcodes for 2008–2009 and 2017–2018 data were categorised using the English Indices of Multiple Deprivation, 2015 for consistency. Index of Multiple Deprivation ranks were used to categorise pupils into five quintiles for analyses (quintile 1 is the most deprived; quintile 5 the least deprived). School is used as a proxy to explore the effect of lunch type: in 2008–2009, School 2 had a mixed take-up of school and home-packed lunches, with substantially more pupils consuming a home-packed lunch; in comparison, School 1 had a higher take-up of school lunches. By 2017–2018, a large majority of pupils in both schools now consumed a school lunch due to the change in policy.

The first analysis considered the effect of change between 2008–2009 and 2017–2018 in mean macro- and micro-nutrients, and key food groups on pupil’s dietary intake at lunchtime only. The second analysis considered the effect of change in mean macro- and micro-nutrients on pupil’s dietary intake in total diet. Linear models were used to explore the effects of principal interest: year of survey, school and the interaction between these two variables at lunchtime and in total diet. All analyses have adjusted for the effect of gender and level of deprivation (Index of Multiple Deprivation). The data for vitamin C were skewed and log transformed prior to analysis; geometric means and ratios are reported. Analyses were conducted in Stata version 15.

## Results

### Summary of participant characteristics and setting

The analyses include 196 pupils with 4 d of dietary data: *n* 112 (2008–2009) and *n* 84 (2017–2018). There were similar percentages of males and females participating in both years (2008–2009: males (*n* 49; 44 %), females (*n* 63; 56 %); 2017–2018: males (*n* 37; 44 %), females (*n* 47; 56 %)). There was no evidence of a difference in level of deprivation between the two time periods (*P* = 0·26). In 2008–2009, the number of pupils having a school lunch was School 1 *n* 24 (92 %); School 2 *n* 26 (30 %). By 2017–2018, post implementation of UIFSM, the majority of pupils consumed a school lunch: School 1 *n* 27/28 (96 %); School 2 *n* 55/56 (98 %); pupils consuming a packed lunch in 2017–2018 were excluded from the analyses (*n* 2). This highlights that in 2008–2009, School 1 had a higher take-up of school lunches; in comparison, School 2 had a mixed take-up of school and home-packed lunches, with substantially more pupils consuming a home-packed lunch. By 2017–2018, a large majority of pupils in both schools now consumed a school lunch due to the change in policy. School 1 is located in the more-deprived ward and School 2 in the less-deprived ward.

### Lunchtime: mean change in macro- and micro-nutrients, and selected food groups

The results of mean change in pupil’s diet at lunchtime are shown in Tables 1–3; Table 1 shows the principal effect of the year and school interaction, and Table 2 shows the effect of year (2008–2009 cf. 2017–2018) and school (School 1 and School 2) independently.

**Table 1:** Lunchtime: the effect of the year and school interaction on mean, mean difference, 95 % CI and *P*-value for %E saturated fat, % E fat, % E NMEs, % E CHO, energy (kJ), food weight (g), Na (mg), Ca (mg), Fe (mg), vitamin C (mg), cakes, fruit and yogurts

	Mean*								
	2008–2009		2017–2018						
	School 1	School 2	School 1	School 2	Mean				
	*n* 112		*n* 84		2008–2009	2017–2018	Difference between difference		
Nutrient/food group	S1 % SL (92); S2 (30)		S1 % SL (96); S2 (98)		S2-S1†	S2-S1	(2017–2018 S2-S1)–(2008–2009 S2-S1)	95 % CI	*P*
% E sat fat	13·5	12·4	5·9	8·3	–1·1	2·4	3·5	1·1, 5·9	0·005
% E fat	31·6	30·3	30·5	32·2	–1·3	1·7	3	–1·0, 7·0	0·14
% E NMEs	12·3	13·4	8·1	8·7	1·1	0·6	–0·5	–4·3, 3·2	0·77
% E CHO	60·5	59·0	57·5	56·4	–1·5	–1·1	0·4	–4·0, 4·8	0·86
Energy (kJ)	2459·4	2003·3	1939·7	1980·3	–456·1	40·6	496·7	231·0, 763·2	< 0·001
Food weight (g)	619·6	444·2	446·5	364·5	–175·4	–82	93·4	29·7, 157·2	0·004
Na (mg)	731·2	605·1	491·3	490·6	–126·1	–0·7	125·4	5·0, 245·7	0·04
Ca (mg)	163·4	201·6	269·0	186·7	38·2	–82·3	–120·5	–178·6, –62·3	< 0·001
Fe (mg)	2·4	2·2	1·9	2·4	–0·2	0·5	0·7	0·4, 1·1	< 0·001
Vitamin C‡ (mg)	110·8	29·5	20·9	15·4	5·2	1·9	2·7	1·7, 4·4	< 0·001
Food groups (portions)									
Yogurts	0·1	0·3	0·2	0·1	0·2	–0·1	–0·3	–0·5, –0·04	0·02

* Mean adjusted for gender and Index of Multiple Deprivation.

† S2-S1 (School 2-School 1).

‡ Geometric means and ratios reported.

**Table 2:** Lunchtime: the effect of year and school independently on mean, mean difference, 95 % CI and *P*-value for % E NMEs, % E fat, % E CHO, vegetables and biscuits (portions)

	Mean*				
	Year†				
	2008–2009 (A)	2017–2018 (B)	Mean difference		
Nutrient/food group	*n* 112	*n* 84	(B-A)	95 % CI	*P*
% E NMEs	13·1	8·5	–4·6	–6·3, –2·9	< 0·001
% E fat	30·6	31·7	1·1	–0·7, 2·9	0·23
% E CHO	59·4	56·6	–2·8	–4·8, –0·8	0·006
Food groups (portions)					
Vegetables	0·4	0·5	0·1	–0·1, 0·3	0·27
Biscuits	0·5	0·1	–0·4	–0·5, –0·3	< 0·001

	School‡				
	School 1 (A)	School 2 (B)			
	*n* 112	*n* 84	(B-A)		
% E NMEs	10·6	11·4	0·8	–3·3, 4·9	0·70
% E fat	30·8	31·1	0·3	–4·0, 4·7	0·87
% E CHO	59·2	57·9	–1·3	–6·1, 3·5	0·60
Food groups (portions)					
Vegetables	0·5	0·4	–0·1	–0·5, 0·4	0·81
Biscuits	0·4	0·3	–0·1	–0·3, 0·04	0·12

* Mean adjusted for gender and Index of Multiple Deprivation.

† Mean adjusted for school.

‡ Mean adjusted for year.

**Table 3:** Total diet: the effect of the year and school interaction on mean, mean difference, 95 % CI and *P*-value for % E saturated fat, % E fat, % E NMEs, % E CHO, energy (kJ), food weight (g), Na (mg), Ca (mg), Fe (mg) and vitamin C (mg)

Nutrient	Mean*								
	2008–2009		2017–2018		Mean				
	School 1	School 2	School 1	School 2	2008–2009	2017–2018	Difference between difference		
	*n* 112		*n* 84		S2-S1†	S2-S1	(2017–2018 S2-S1)–(2008–2009 S2-S1)	95 % CI	*P*
% E sat fat	15·1	13·5	10·6	12·1	–1·6	1·5	3·1	1·6, 4·5	< 0·001
% E fat	33·4	31·2	32·0	31·9	–2·2	–0·1	2·1	–0·2, 4·4	0·08
% E NMEs	15·9	14·7	11·6	10·9	–1·2	–0·7	0·5	–2·2, 3·3	0·71
% E CHO	57·9	58·1	56·6	57·2	0·2	0·6	0·4	–2·2, 3·1	0·75
Energy (kJ)	6294·8	6125·4	6004·5	6181·9	–169·4	177·4	346·8	–366·1, 1059·4	0·34
Food weight (g)	1508·7	1528·7	1457·7	1531·0	20	73·3	53·3	–156·3, 263·0	0·62
Na (mg)	1969·8	1949·1	1830·5	1881·5	–20·7	51·0	71·7	–211·8, 355·2	0·61
Ca (mg)	704·3	772·1	721·5	796·1	67·8	74·6	6·8	–136·8, 150·5	0·93
Fe (mg)	5·9	7·7	6·7	8·0	1·8	1·3	–0·5	–1·6, 0·7	0·41
Vitamin C‡ (mg)	158·5	100	79·4	63·1	2·0	1·3	1·6	1·2, 2·1	0·004

* Mean adjusted for gender and Index of Multiple Deprivation.

† S2-S1 (School 2-School 1).

‡ Geometric means and ratios reported.

#### Principal effect: year and school interaction (macro- and micro-nutrients)

For several nutrients examined, we found evidence of a statistically significant year (2008–2009 and 2017–2018) and school (School 1 and School 2) interaction and the subsequent effect on pupils’ mean intakes (Table 1). We found evidence of a year and school interaction on pupils mean intakes as food weight (g) and from percentage energy saturated fat, energy (kJ), and from Na (mg), Ca (mg) and Fe (mg). In 2008–2009, pupils in School 2 had a slightly lower mean intake of percentage energy from saturated fat than pupils in School 1 (–1·1 %); by 2017–2018, pupils in both schools had a lower mean intake, but in 2017–2018 pupils in School 2 now had a higher mean intake from percentage energy saturated fat than pupils in School 1 (2·4 %; difference in difference: 3·5 %; 95 % CI 1·1, 5·9; Table 1). In 2008–2009, pupils in School 2 had a lower mean energy (kJ) intake than pupils in School 1; by 2017–2018, pupils in School 1 now had lower mean energy (kJ) intakes and of a similar intake to pupils in School 2 (Table 1). In 2008–2009, pupils’ mean Na intakes were lower in School 2 than School 1, mean Ca intakes were higher, while mean Fe intakes were similar. By 2017–2018, pupils’ mean Na intakes were now similar in the two schools, mean Ca intakes were now higher in School 1 and while mean Fe intakes were now higher in School 2, they were lower in School 1 (Table 1). We found no evidence of a statistically significant interaction for percentage energy from fat, NMEs and CHO.

#### Principal effect: year and school interaction (food groups)

**Fig. 1:**
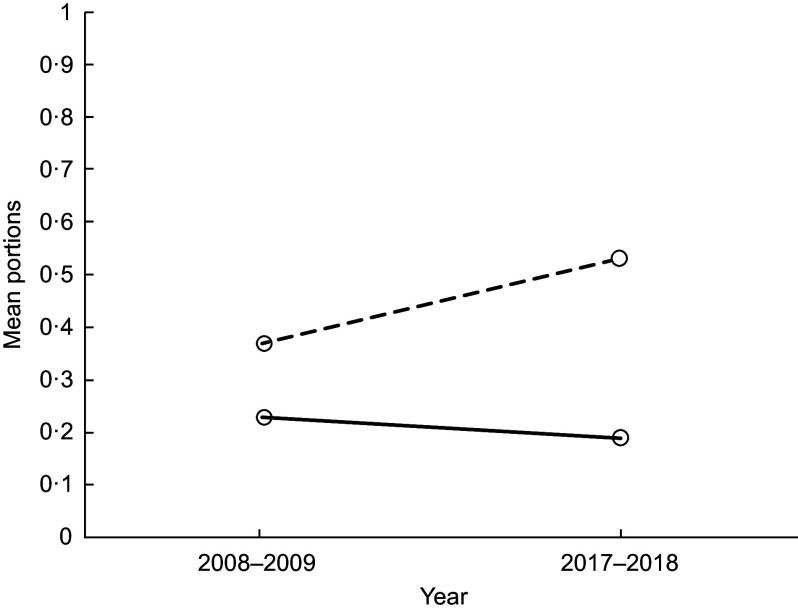
Lunchtime: the effect of year and school on mean portions of cake/sweet pudding. (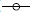), School 1; (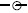), School 2

**Fig. 2:**
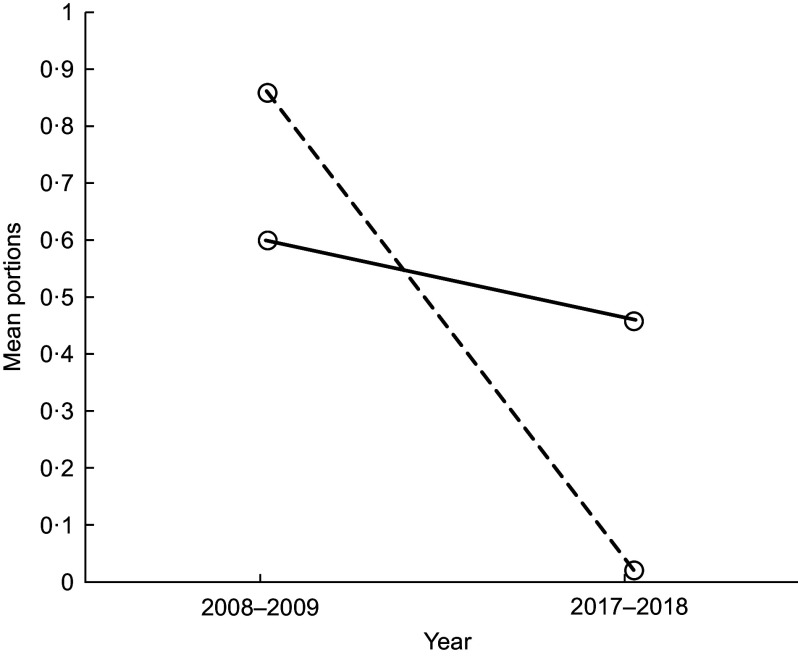
Lunchtime: the effect of year and school on mean portions of fruit. (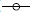), School 1; (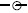), School 2

We found evidence of a year and school interaction on pupils’ mean portions of cakes/sweet puddings, fruit and yogurts (Figs 1 and 2 and Table 1, respectively). In 2008–2009, mean portions of cakes/sweet puddings were similar in both schools; by 2017–2018, pupils in School 2 had a higher mean portion intake of cakes/sweet puddings than pupils in School 1 (Fig. 1). Between 2008–2009 and 2017–2018, mean portions of fruit intake remained similar in School 1 but were now lower in School 2 (Fig. 2). This was similar for mean portions of yogurt (Table 1).

#### The effect of year (macro- and micro-nutrients, and food groups)

There was evidence that between 2008–2009 and 2017–2018 mean percentage energy from NMEs was lower (mean change –4·6 %; 95 % CI –6·3, –2·9) and mean percentage energy from carbohydrate (CHO) was lower (Table 2). We found no evidence of a difference in mean percentage energy from fat; while mean portions of biscuits were lower, there was no evidence of a change in mean portions of vegetables (Table 2).

#### The effect of school (macro- and micro-nutrients, and food groups)

We did not find evidence of a school affect on mean percentage energy from NMEs, fat, CHO, mean portions of vegetables or biscuits (Table 2).

### Total diet: mean change in macro- and micro-nutrients

The results of mean change in pupil’s total diet are shown in Tables 3 and 4. Table 3 shows the principal effect of the year and school interaction, and Table 4 the effect of year (2008–2009 cf. 2017–2018) and school (School 1 and School 2) independently.

**Table 4:** Total diet: the effect of year and school independently on mean, mean difference, 95 % CI and *P*-value for % E NMEs, % E fat, % E CHO and absolute energy (kJ), food weight (g), Na (mg), Ca (mg) and Fe (mg)

	Mean*				
	Year†				
	2008–2009 (A)	2017–2018 (B)	Mean difference		
Nutrient	*n* 112	*n* 84	(B-A)	95 % CI	*P*
% E NMEs	14·9	11·1	–3·8	–5·2, –2·7	< 0·001
% E fat	31·7	31·8	0·1	–0·9, 1·2	0·80
% E CHO	58·0	57·0	–1·0	–2·2, 0·1	0·08
Energy (kJ)	6163·9	6125·0	–38·9	–358·6, 280·7	0·81
Food weight (g)	1522·3	1510·0	–12·3	–106·2, 81·5	0·80
Ca (mg)	753·7	775·8	22·1	–42·2, 86·4	0·50
Fe (mg)	7·2	7·7	0·5	–0·1, 1·0	0·08
Na (mg)	1953·3	1866·0	–87·3	–214·3, 39·6	0·18

	School‡				
	School 1 (A)	School 2 (B)			
	*n* 112	*n* 84	(B-A)		
% E NMEs	14·0	13·1	–0·9	–3·9, 2·0	0·53
% E fat	32·5	31·5	–1·0	–3·5, 1·6	0·45
% E CHO	57·3	57·7	0·4	–2·4, 3·3	0·78
Energy (kJ)	6125·8	6155·9	30·1	–743·1, 803·3	0·94
Food weight (g)	1480·1	1530·8	50·7	–176·4, 277·7	0·66
Ca (mg)	710·6	782·4	71·8	–83·7, 227·3	0·36
Fe (mg)	6·3	7·8	1·5	0·24, 2·7	0·02
Na (mg)	1901·6	1922·1	20·5	–286·5, 327·6	0·89

* Mean adjusted for gender and Index of Multiple Deprivation.

† Mean adjusted for school.

‡ Mean adjusted for year.

#### Principal effect: year and school interaction (macro- and micro-nutrients)

We found evidence of a year and school interaction on pupils’ mean intakes from percentage energy saturated fat. In 2008–2009, pupils in School 2 had a lower mean intake than pupils in School 1 (–1·6 %); by 2017–2018, pupils in both schools had a lower mean intake, but now pupils in School 2 had a higher mean intake from percentage energy saturated fat than pupils in School 1 (mean difference 1·5 %; difference in difference: 3·1 %; 95 % CI 1·6, 4·5; Table 3). In 2008–2009, pupils in School 1 had a higher mean intake of vitamin C than those pupils in School 2; by 2017–2018 although mean intakes of vitamin C were lower in both schools, they were now similar (Table 3). We found no evidence of a statistically significant interaction for the remaining nutrients presented in Table 3.

#### The effect of year (macro- and micro-nutrients)

We found evidence that between 2008–2009 and 2017–2018, mean percentage energy intake from NMEs in pupil’s total diet was lower (mean difference –3·8 %; 95 % CI –5·2, –2·7). We found no other evidence of a year effect (Table 4).

#### The effect of school (macro- and micro-nutrients)

We found limited evidence that school had an effect on pupil’s total dietary intake (Table 4).

## Discussion

In 2008–2009, School 1 had a higher take-up of school lunches; in comparison, School 2 had a mixed take-up of school and home-packed lunches, with substantially more pupils consuming a home-packed lunch. By 2017–2018, a large majority of pupils in both schools now consumed a school lunch due to the change in policy. We found some positive changes that could potentially improve pupil’s diet; key findings are summarised here.

By 2017–2018, although pupils in both schools had a lower mean percentage energy intake from saturated fat, now pupils in School 2 had a higher mean intake than pupils in School 1; this was also reflected in total diet at lunchtime; by 2017–2018 pupils’ mean Na intakes were now similar in the two schools, this may be due to a change from home-packed lunches which often contain crisps to school lunches. The reduction in both mean % E saturated fat and Na is small but important reductions in improving children’s dietary intakes.

With regard to the food choices pupils made, we found a decrease in mean portions of biscuits between 2008–2009 and 2017–2018. However, we found some changes which were less encouraging. In 2008–2009, mean portions of cakes/sweet puddings were similar in both schools, but by 2017–2018 pupils in School 2 had a higher mean portion of cakes/sweet puddings than pupils in School 1, and the difference between schools increased. Likewise, while mean portions of fruit and yogurt remained similar in School 1 between 2008–2009 and 2017–2018, these decreased in School 2. The options after the main meal for school lunch were cake/sweet pudding, biscuits, fruit (usually whole) and yogurt. These options are compliant with the food-based standards in both schools. However, these findings highlight that unsurprisingly the daily availability of cake/sweet pudding increases the option for pupils to choose this. School 2 had all these options, on all days during the data collection period; School 1 had 1 d where pupils could only choose fruit. While both schools are compliant with the food-based standards, the headteacher in School 1 chose to restrict the daily choice of a pudding and to have a day where this option was removed and only fruit could be selected. Although pupils in School 1 may not have chosen the fruit, they were unable to choose a cake/sweet pudding. This potentially explains the limited change in fruit and cakes/sweet pudding in School 1. In contrast, pupils in School 2 decreased their fruit intake. This may be due to the change from home-packed to school lunch and not choosing fruit if cakes/sweet puddings and biscuits are an alternative choice. These findings emphasise combined policy and environment approaches are together likely to have a more positive effect on pupil’s food choices. There is increasing attention in public health to enable the ‘*healthy choice’* to be *‘the easy choice’*^(24,25)^. Part of this approach could be to remove the less healthy choice, such as the daily option of cake/sweet puddings in schools. This approach is supported by Guthrie *et al.* who advocate removing less nutritious foods in schools to encourage pupils to select healthier options^(26)^.

At lunchtime, pupils’ mean percentage energy NMEs was lower in 2017–2018 compared with 2008–2009. The potential impact of the UIFSM policy on pupil’s total dietary intake was mainly limited to % E NMEs. Although mean intakes remain above recommendations, mean intakes of % E NMEs have significantly reduced; this is a positive finding to improve children’s dietary intake. In 2017–2018, all pupils included in these analyses consumed a school lunch. This was a notable change from 2008 to 2009 where the majority of pupils in School 2 consumed a home-packed lunch. A change from home-packed to school lunch involves a change in the food and drink choices of pupils. The decrease from %E NMEs from 2008 to 2009 and 2017 to 2018 may be explained by the change from home-packed to school lunch. Prior research found home-packed lunches to contain a higher NMEs content^(19,27)^.

Key limitations were only two schools were included limiting the generalisability of results, there was no control group and we used repeat cross-sectional studies. Pupils in both schools were recruited using opt-in where parents had to actively consent for their child to participate; therefore, the sample may not be representative of the total population. Level of deprivation did not differ in the participants between the two time points. It is reasonable to suggest pupils in School 1 may have been more likely to be in receipt of Free School Meals in 2008–2009, although we do not have this level of Free School Meals status at a pupil level. As with all dietary surveys, there are limitations with the dietary assessment methods. The dietary data collection method used in the current study employs average food and drink portions that are age and sex specific as noted in the methods; therefore, individual-level child variation in portion weight is not reflected in the findings. Menu cycles in schools are based on a 3-week cycle; foods offered and chosen by pupils are potentially different, this may impact on the nutritional content and findings. A key strength was the availability of dietary data from Key Stage 1 pupil’s pre-implementation of the UIFSM policy. This enabled a natural experimental design to evaluate the impact of UIFSM on pupil’s dietary intake at school and in their total diet pre- and post-implementation. Despite only two schools participating, there are approximately 4 d of dietary data from 200 pupils to explore this change in policy across the socio-economic spectrum. Within the limitations, the current study uniquely provides insights into the impact of UIFSM on pupil’s dietary intake.

There is potential for such large-scale policy changes to have positive (i.e., improving dietary intake^(13,28)^) and/or negative effects (i.e., widening health inequalities^(29)^). Prior to implementing large-scale public health policies, careful planning to evaluate implementation and the effectiveness needs to be considered by policy-makers. Within the limitations of this pilot study, these findings indicate both positives and limitations following the implementation of UIFSM on pupil’s diets and have shown the role of school-level food practices on pupil’s food choices. To facilitate maximum potential of UIFSM on pupil’s diets, national levers, such as the current discussion on the new school food standards relating to sugars^(25)^ could consider removing the traditional daily ‘sweet pudding’ option and advocating ‘fruit only’ options on at least 1 d/week, as some schools do already. Small school-level changes, such as ‘fruit only’ days, could potentially maximise positive health impacts of the food-based standards and implementation of UIFSM by decreasing NMEs intake. As noted, one of the limitations of this pilot study was a small sample size in two schools in the NE of England. A more robust evaluation of this policy is imperative to consider adherence to the policy, whether pupils participate in the UIFSM programme, the impact on pupils diet at both lunchtime and their total diet, the equitability across the socio-economic spectrum, the wider effects on schools and families and cost-effectiveness.
